# Co‐creation of information materials within the assent process: From theory to practice

**DOI:** 10.1111/hex.13675

**Published:** 2022-11-23

**Authors:** Jaime Fons‐Martinez, Cristina Ferrer‐Albero, Javier Diez‐Domingo

**Affiliations:** ^1^ Vaccine Research Area Foundation for the Promotion of Health and Biomedical Research of Valencia Region, FISABIO Valencia Spain; ^2^ Facultad de Medicina y Ciencias de la Salud Universidad Católica de Valencia San Vicente Mártir Valencia Spain

**Keywords:** assent, design thinking, ethics, information materials, informed consent process, participant‐centred design

## Abstract

**Introduction:**

The informed consent process is key to safeguarding the autonomy of the participant in medical research. For this process to be valid, the information presented to the potential participant should meet their needs and be understood by them. The i‐CONSENT project has developed ‘Guidelines for adapting the informed consent process in clinical trials’ which aim to improve informed consent so that they are easier to understand and better adapted to the needs and preferences of the target population. The best way to tailor information to the characteristics and preferences of the target population is to involve the community itself.

**Methods:**

Following guidelines developed by i‐CONSENT, assent materials were co‐created for a mock clinical trial of the human papillomavirus vaccine in adolescents. During the process, two design thinking sessions were conducted involving a total of 10 children and 5 parents. The objectives of the sessions were to find out the children's opinion of the informed consent (assent in their case) process in clinical trials, identify the parts that were most difficult to understand and alternatives for their presentation and wording, identify the preferred formats for receiving the information and the main characteristics of these formats, design a video explaining the clinical trial and evaluate a tool for assessing comprehension.

**Results:**

Assent materials were co‐created in three formats: a web‐based material following a layered approach; a video in story format; a pdf document with an innovative way of presenting information compared to traditional assent documents. In addition, the Comprehension of Assent Questionnaire was co‐designed, based on the Quality of Informed Consent questionnaire.

**Conclusion:**

The design thinking methodology has proven to be an easy and useful tool for involving children in designing information tailored to their needs and preferences.

**Patient or Public Contribution:**

A sample of the target population participated in the design and piloting of the materials created using design thinking methodology. In addition, patient representatives participated in the design and evaluation of the guidelines developed by the i‐CONSENT project that were followed for the development of the materials in this study.

## 1 INTRODUCTION

Many people still believe that the term informed consent (IC) is limited solely to obtaining the signature of research participants in the Informed Consent Form (ICF), unaware that this act is part of a much broader Informed Consent Process (ICP).^1^

During the ICP, efforts are made to protect the rights and welfare of participants at all times. The right to health protection is the main objective of legislators, researchers, sponsors, health professionals and the pharmaceutical industry. But the right to justice, freedom and participant autonomy must be ensured in all research involving human subjects.^2^

The ICP, described step‐by‐step in the ‘Guidelines for tailoring the Informed Consent Process in Clinical Studies’,^3^ focuses on a continuous bidirectional communication process between the participant and the research team. It starts at the first contact of the potential participant with the study and continues until the end of the study and the corresponding dissemination of its results.^4^

There are therefore a series of phases in which relevant information is provided from the first contact with the potential participant. This information is discussed and clarified in an interview with a member of the research team who is trained to perform competently and with integrity.^1^ The decision on whether or not to participate in the study should be made after ensuring that the potential participant has understood all relevant information provided and that any doubts that may have arisen have been resolved.

The central axis of the whole process is the relationship that is created between the researcher and the study participants. Knowledge, empathy, active listening, communication skills and respect should not be lacking in this relationship.

But since the interpersonal relationship that is created is not traceable and no record of what is discussed or talked about can be kept, it is necessary to ensure that the relevant information from any research study is presented and available to the potential participant in a clear, concise and patient‐friendly manner.

The best way to adapt it to the characteristics and preferences of the target population is to involve the community itself, or a representative group of the community, in the design, development and execution of the ICP monitoring of the research, as well as in the dissemination of the results.^1^

In the same way that lay members are included in Ethics Committees to provide that perspective of potential participants, inviting lay members or patient groups to participate in the development of IC materials and resources will have a positive impact on the end result, as the process will be better understood and more suited to potential participants. Industry and patient organizations are committed to improving collaboration and building trust with all parties involved. The document developed by the European Federation of Pharmaceutical Industries and Association (EFPIA) on how to work with patient groups^5^ is a reference point to guide these interactions.

This is the result of a shift from the traditional paternalist paradigm of care, inherited from Hippocratic medicine to a patient‐ and family‐centred paradigm of care.

One of the first initiatives in this direction was the creation of Patient‐Focused Medicine Development (PFMD) in 2015,^6^ whose mission was to bring together and include all healthcare stakeholders in an open coalition for shared decision‐making and to provide healthcare solutions. Among the outcomes of this collaboration, a practical guide was developed^7^ for planning, developing and evaluating the quality of patient involvement activities and projects in the development and lifecycle of medicines.

Between 2012 and 2017, the European Patients' Academy on Therapeutic Innovation (EUPATI)^8^ project was developed with the aim of increasing patients' involvement in the development and research of new medicines and treatments, improving their health literacy, becoming patient experts and empowering them in the management of their own health.

In the field of rare diseases, the Share4Rare project launched in 2018,^9^ and seeks to empower patients by increasing their knowledge through information materials created in collaboration with patients.

With the aim of developing guidelines to help improve the ICP, the i‐CONSENT project was launched in 2017.^4^ One of the key points of the project is the inclusion of potential participants in the design and review of the information materials in a research setting, to ensure that they are understandable and tailored to the needs and preferences of the target population.^3^

Balik's^10^ approach to providing patient‐ and/or family‐centred care envisages three different approaches: ‘doing to’, ‘doing for’ and ‘doing with’. When we apply this to the ICP, we are faced with the challenge of making IC materials with the patient, where potential participants are involved in all phases of the process, especially in the design of information materials. To do this, sponsors and researchers must first understand the target population and then incorporate them into the design, development and review of the information materials to make them more inclusive and tailored to the actual needs of the participants.^3^

Tool V proposed in the guidelines, entitled ‘Methodologies and tools to incorporate the participants' perspective’,^3^ proposes design thinking and focus group methodology to identify problem areas in the IPC, define and prioritize these problems and develop joint ideas and prototypes to solve them.

The participant is thus an active part of scientific progress and not a passive research subject. Co‐creation in the ICP within any study seeks to encourage fair and open participation and quality input based on the experience and expertise of all stakeholders.

This article describes the process of developing informational materials for a hypothetical clinical trial (CT) with children following the recommendations of the i‐CONSENT project. It focuses on the description of strategies for the co‐creation of materials based on the characteristics of the target population, their needs and preferences.

## 2 METHODS

Taking for granted the social and scientific value that any research must have to be carried out, we worked on the design and co‐creation phase of the information materials for a simulated study, following the recommendations of the i‐CONSENT guidelines.^3^ The steps to be followed in the development of materials are summarized in Table 1.

**Table 1:** Points to consider when preparing study information

☐ Have information materials been prepared taking into account the target population?
☐ Have you tested your communication materials with representatives of your target population? Have you tested it with men and women (if applicable)?
☐ Is the information clear and concise?
☐ Is the information relevant and complete?
☐ Has it been presented in a neutral/balanced way?
☐ Have you provided references to reliable sources of information?
☐ Does the study include placebo control? Have you informed participants about the details of its use and the placebo effect?
☐ Have you informed participants about incidental findings policy?
☐ Have you considered a range of media channels/platforms/formats?
☐ Have all the information materials been approved by an Independent Ethics Committee?

The scenario for the assent materials is that of the human papillomavirus (HPV) vaccine CT in adolescents, taking into account gender differences.

The target population and the scenario were defined according to the i‐CONSENT project study protocol,^4^ considering healthy children aged 12–13 years old for participation. In the same way and following the same protocol, the result of the co‐creation work of information materials was validated in a later phase, measuring their comprehension in Romania, Spain and the United Kingdom. It was therefore necessary to create an information comprehension assessment tool.

The technique chosen to work with the target group was ‘design thinking’,^11^, ^12^, ^13^, ^14^, ^15^ as it is a directly user‐centred, action‐oriented technique aimed at generating innovative solutions to a given problem. It involves several phases: empathizing, defining, devising, prototyping and validating or testing.

### 2.1 Development of the design thinking sessions

Two face‐to‐face sessions were scheduled in Valencia, Spain. Recruitment was done through the paediatric network VIVA (Vaccine Institute of Valencia), together with members of the i‐CONSENT team. Participants were boys and girls aged 12–13 years, with no previous experience of participating in CTs and in good health. This is a challenge for vaccine CTs, as participants have no experience with the disease and are not aware of the indirect benefit of their participation.

As the aim of the sessions was to prepare materials that could be useful and easy to understand for both those who have previously participated in CTs and have knowledge of the terminology and processes used in them, and those who have never participated in this type of research, it was decided to include only participants with no previous research experience, since they are the ones who, in principle, are at a disadvantage in understanding and have the greatest need for information. It was also considered that there may be a risk that those who had already participated in CTs could monopolize the conversation and make the rest of the participants uncomfortable because they were unfamiliar with certain terminology or processes. Convenience sampling was used, where three paediatricians from the VIVA network offered participation to parents and children in the consultation. Those who showed interest in participating voluntarily were invited to contact the i‐CONSENT research team. All participants were informed of the purpose of the sessions, the benefit to other children, the inconvenience their participation might entail in terms of time and travel, the protection of their data and the right to withdraw at any time without giving any reason. They gave their assent to participate, and the parents gave their consent. A total of 10 children participated in the design sessions.

To create a safe and open space to increase comfort, trust and participation, the following strategy was applied:

(1) Sessions began with group dynamics focused on: introducing the participants and the researchers; informing them that other children had participated or were going to participate in similar sessions; highlighting the importance of each participant's role in the research, making them feel that a diversity of opinions among the participants was welcome and that all contributions were important to us.
(2) Many of the activities included written expression, with subsequent reading aloud by the researcher. This meant that an idea or answer was not attributed to any specific person, encouraged all opinions to be heard no matter who said it and prevented the exercise from being monopolized by any one participant.

### 2.2 First session

The objectives of the first session were:

(1) Create a climate of trust and empathy between children, parents and the research team.
(2) Share views on CTs for vaccine development and identify wishes and needs relevant to the group of participants and their parents.
(3) Prototype assent materials with preferred formats.

Two members of the research team welcomed the five children and their five parents and acted as facilitators guiding the group through the process. The participants were introduced to each other using a dynamic presentation through a game with a ball to encourage interaction between them. With this playful component, a positive emotional climate was established and the relaxation of those involved was achieved.

As this was a group of healthy children with no previous experience of participating in CTs, and in order for them to understand what a CT is, a 5‐min 11‐s educational video in Spanish on how a CT is developed and conducted, produced by the European Communication on Research Awareness Needs (ECRAN),^16^ was shown. The aim was to understand what would be really relevant for children and parents if they would participate in a CT with vaccines.

Subsequently, a role‐play was conducted with a vaccine CT scenario, in which both children and parents participated by assuming a role (participant, parents, researcher or doctor) and following a given script. At the end of the role‐play, participants were given a traditional assent form to read and make decisions. They were given the paper‐based assent document, based on the ICF used in a real trial (EudraCT no. 2006‐000764‐85) and were given the time they needed to read it.

Participants expressed their emotions, using balloons on which they drew faces expressing their mood with the information received in the assent and how they would feel if they had to make the decision to participate in the CT at that moment. In this way, it was possible to better understand the problems experienced by the participants and the feelings they have in a situation such as this.

With the information obtained the focus of action could be defined by focusing on the aspects relevant to the participants. The format ‘The (user) wants/needs (want/need) because (insight)’ was used.

The information collected was clustered into different areas of improvement: information (purpose, risks, benefits, personal data, right to revoke, conditions, procedure), format (web, app, video, comic, text, oral explanation) and decision‐making (individual, shared).

Once the focal points for action had been collected and synthesized, the question arose as to how we could devise and design the best solutions to the problems raised.

To this end, through brainstorming, participants reflected on the information presentation formats they would prefer and were asked to design a prototype of assent material (video and infographic).

With all this work (summarized in Table 2), the first session ended and their participation was thanked.

**Table 2:** Objectives and methodology for the first design thinking session with children and their parents

Objectives	Methodology
Empathize	Presentation dynamic: ‘passing the ball’
Identify and define	Viewing video on Clinical Trials
	Vaccine clinical trial role play and decision making with a traditional text‐based reporting document
	Clustering to define areas for improvement: information and formatting
Devising	Brainstorming for alternative presentation formats
Designing prototypes	Design of prototypes with different formats (video and infographics)

### 2.3 Second session

The second design thinking session included more detailed tasks involving another five children at the same age. The objectives were different, as the results of the previous session were already being used as a starting point:

(1) Detect words that are difficult to understand, and propose a glossary of terms.
(2) Read the modified written assent document for the hypothetical HPV vaccine study to identify information that is difficult to understand and propose a plain language explanation.
(3) Evaluate the comprehension assessment tool.
(4) Assess the understanding of the information provided.

The second session began with a review of the previous session in the form of a narrative story, telling them about when and where the previous session took place, the characteristics of the children who participated, the objectives of the session and the results obtained. The points for improvement identified in session 1 were presented on a whiteboard using a mind map. This allowed to focus the children's attention, introduce them to the topic and the progress of the first session and explain the objectives of the second session.

The mind map graphically represented the main ideas, highlighting the most relevant points and making it easier for the children to focus their attention and follow the story. The first area of improvement detected in the previous session referred to the amount of information included in the initial document. Following the guidelines set out in Fact Sheet IV of the i‐CONSENT guide: ‘Information to be given to potential participants during the information phase’ and taking into account the EU 536/2014 Regulation on CTs,^17^ the original information document worked on in the first session was adapted.

The title proposed as a result of the text adaptation was: ‘Phase III study on the HPV vaccine in youth from 9 to 14 years of age’. The i‐CONSENT guidelines recommend using inclusive language and avoiding gendered roles. We also followed the recommendations on the gender perspective included in the guidelines, which recommends developing a single material for all participants, in the event that there are no exclusion criteria based on gender; and the recommendations to adapt the information to the minor's age and maturity.

As the amount of information in the text document proved to be overwhelming in the first session, the information was presented using a layered approach, maintaining the completeness of the information provided. The first layer was prepared with the relevant information, and the second was left for further information and a glossary of terms difficult to understand.

To test the new assent document prepared for the second session, the participants were asked to mark in colour the words they did not understand. Members of the research team explained the terms they did not understand, and the participants were asked to write an explanation in their own words. The definitions were accompanied by their own illustrations, which provided guidance on the type of drawing and the aspects to be highlighted.

Thus, a glossary of terms difficult to understand was created with the participants to expand the information in plain language and use it in a second layer with additional information. It included the concepts of a placebo, vaccine safety, blood tests, confidentiality and the right of revocation.

In terms of format, as requested in the first session, the use of graphic components to complement the information such as icons, infographics and simple and easy‐to‐interpret images was added, making the written information more easily readable and understandable.^17^

The use of digital tools and/or multimedia components^18^ and the possibility of offering the participants different formats to receive the information was worked on with the children. In both sessions, the four options most discussed were: text, video, comic and web. Through brainstorming, the children contributed their preferences and then worked on a prototype of a website to present the information.

It is important to consider the provision of information in written or digital format as a complement to, not a substitute for, face‐to‐face discussions with the research team. Evidence suggests that simple and brief consent forms, accompanied by a meaningful conversation between participants and researchers, can improve comprehension.^19^

To assess comprehension of the information, an Assent Comprehension Questionnaire for vaccine studies (abbreviated ‘C‐CAsIn’ for ‘Cuestionario de Comprensión del Asentimiento Informado’) was developed in Spanish, based on the Quality of Informed Consent (QuIC).^20^

During this session, the comprehension of the items of the C‐CAsIn questionnaire was analysed. Those items that raised doubts were rewritten with the children's help. The Likert‐type response was adapted by changing the numbers (1–5) with small icons that graphically represented an emotion or idea (emoticons).

**Figure 1:**
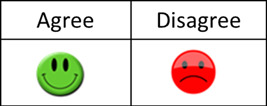
Possibilities of response for the objective part (Part A) of the C‐CAsIn. C‐CAsIn, Comprehension of Assent Questionnaire.

In the first part of the questionnaire, which assesses comprehension objectively, the response possibilities for each statement were represented by a green, smiling icon for ‘agree’ and a red, sad icon for ‘disagree’ (see Figure 1).

**Figure 2:**
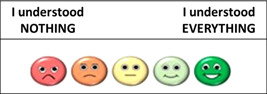
Possibilities of response for the subjective part (Part B) of the C‐CAsIn. C‐CAsIn, Comprehension of Assent Questionnaire.

In the second part of the questionnaire, which assesses comprehension subjectively, the response possibilities were widened and broken down further, with the possibility of choosing between five degrees of comprehension between ‘I understood NOTHING’ and ‘I understood EVERYTHING’ (see Figure 2).

The last part of the questionnaire includes a series of general questions about previous experience in a CT, satisfaction with the information received, the preferred format for receiving the information and sense of understanding of all the information.

Before closing the session, a brainstorming session was held on how to improve the information received, how they would adapt it to an interactive format and what elements they would use to support the information (links, pop‐ups, embedded videos, etc.). Table 3 summarizes the work done during the second session.

**Table 3:** Objectives and methodology of the second design thinking session with children

Objectives	Methodology
Empathize	Narrative story and mind map explaining the previous session and placing the main issue in the centre (information in assent) and connecting the different strands or areas of improvement: information and format
Identify and define	Reread adapted information document design to identify poorly understood concepts and define glossary of terms for second layer of information
Designing prototypes	Web prototype design Brainstorming: features of narrated video
Validate/test	Test the assent comprehension assessment questionnaire
	Test the information received

**Table 4:** Comprehension of Assent Questionnaire (C‐CAsIn) Part A

No.	Question	Agree	Disagree	Section of information
A1	I can decide to participate in this study without discussing it with my parents. Their opinion does not matter.	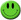	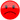	Decision‐making
A2	One of the benefits of participating in this study is helping other children. What the researchers learn from me can be applied to others.	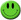	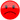	Indirect benefit
A3	The researchers have told me how long the study will take.	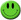	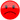	Procedures
A4	The study vaccine has been tested before in many girls and boys.	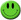	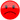	Procedures
A5	One of the objectives of this study is to see how safe the vaccine is.	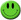	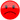	Aim of the study
A6	One of the benefits of participating in this study could be improving my defenses against diseases.	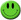	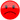	Direct benefit
A7	After I decide to participate in this study, I will be randomly (like playing heads or tails) put in a group.	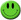	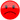	Randomization procedure
A8	I will know what group I am put in throughout the whole study.	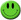	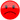	Blinding Procedures
A9	If I receive the placebo, my defenses will improve.	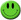	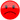	Placebo Procedures
A10	Participating in this study does not involve any risk or inconvenience.	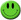	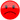	Risks
A11	By participating in the study, I would be helping the investigators to know more about the product they study.	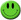	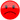	Aim of the study
A12	The information that I have read explains who I have to talk to if I am worried or if I have any questions.	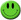	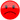	Further information
A13	If I do not want to participate, I can leave the study without any problem.	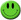	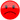	Voluntariness
A14	I have to stay in the study even if I want to quit.	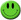	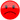	Right to withdraw

**Table 5:** Comprehension of Assent Questionnaire (C‐CAsIn) Part B

Num.	I understood…	I did not understand ANYTHING	I understood EVERYTHING
B1	That the study vaccine is being investigated.	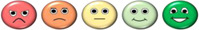	
B2	That my participation in the study will help other children.	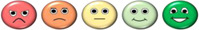	
B3	How long will I be in the study.	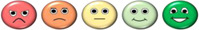	
B4	What the researchers are trying to achieve by doing this study.	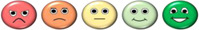	
B5	What will be done at each visit.	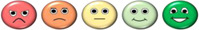	
B6	The possible risks and inconveniences of participating in this study.	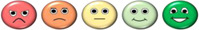	
B7	The possible benefits of participating in the study.	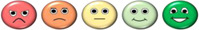	
B8	Which people will know that I am participating in the study.	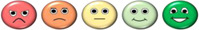	
B9	Whom I will need to talk to if I have any questions or worries about the study.	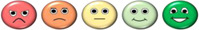	
B10	That it is not compulsory for me to participate in this study.	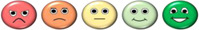	

## 3 RESULTS

Ten healthy children with no previous experience in CTs and their parents participated in the design thinking sessions. All the children were 12–13 years old and lived in the Valencian Region.

The final design of the assent information materials for the hypothetical trial with minors was discussed with external design and digital communication experts.

The text was improved in terms of its linguistic readability using the Fernández‐Huerta Index (IFH)^21^ and the Flesch‐Szigriszt Index (INFLESZ) readability scale,^22^, ^23^ using the web tool ‘Legible’.^24^ The full‐text readability scores of the first layer were:

(1) IFH: ‘easy’ (80.46 points);
(2) INFLESZ index: ‘fairly easy’ (76.52 points);
(3) Estimated reading time: 6 min;
(4) Years of schooling needed to understand Crawford's^25^ formula: 4 years.

Following the suggestions of the children, visual aids were added and the text was accompanied by images, animated gifs and photographs featuring children.

**Figure 3:**
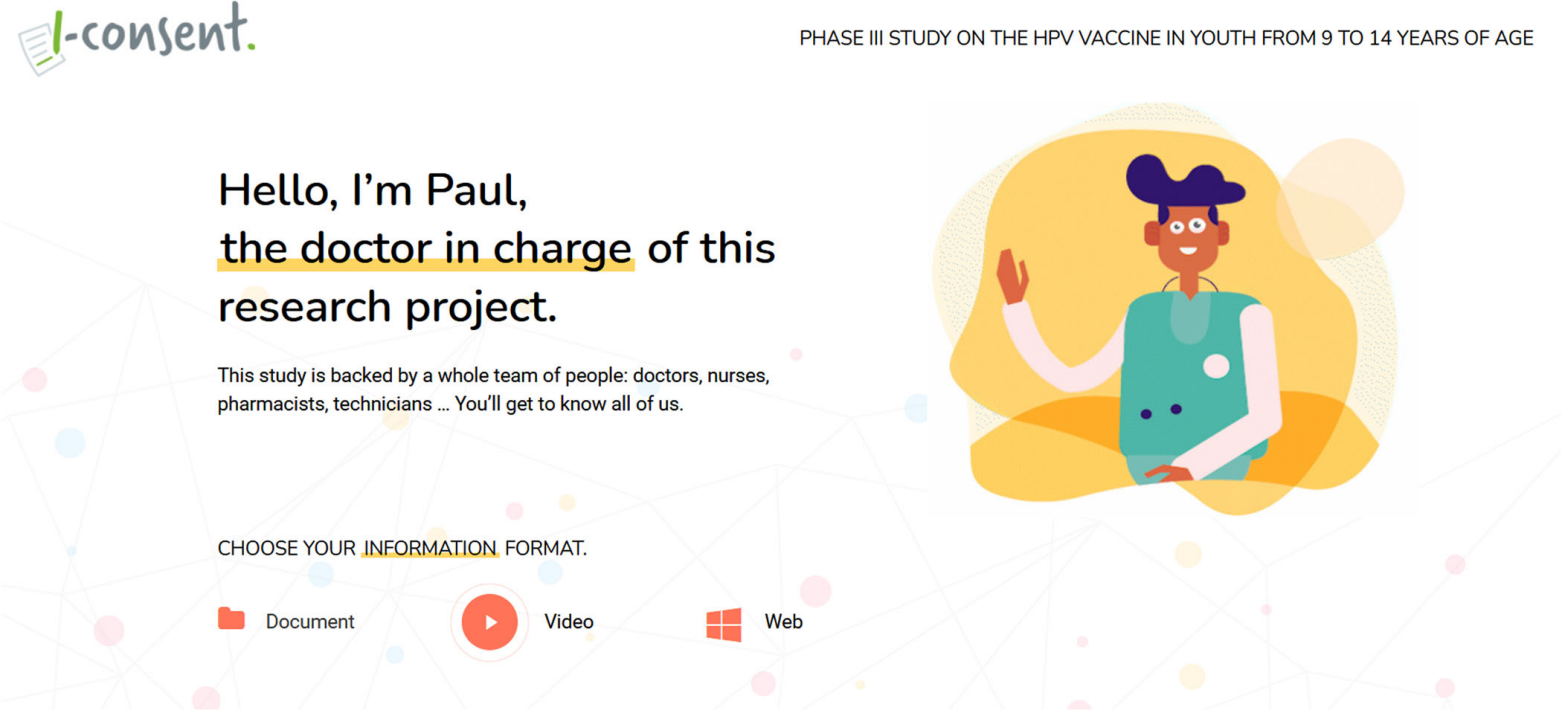
Screenshot of the final materials (http://iconsent.pilotvalidation.eu/en/teenagers-study/)

**Figure 4:**
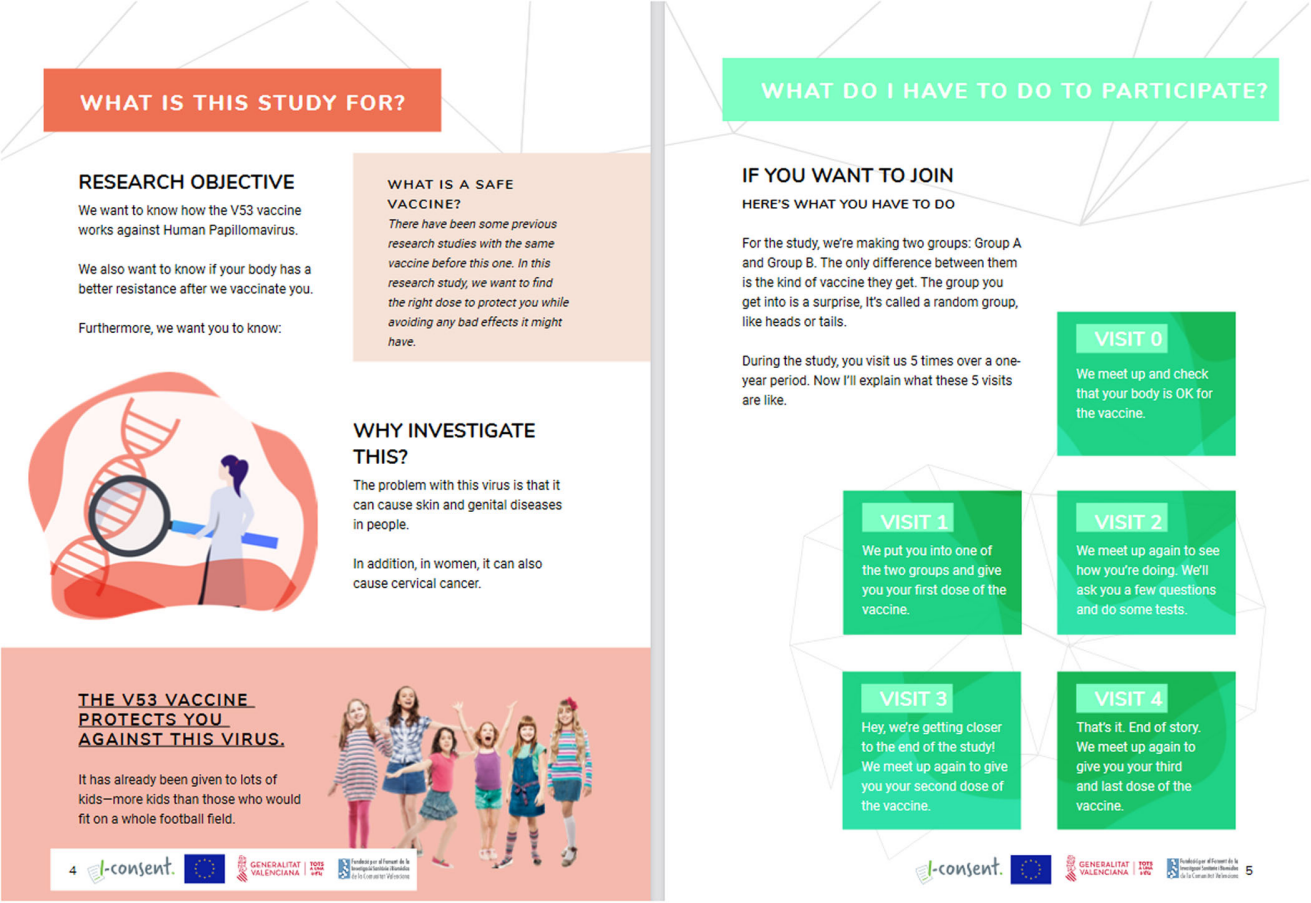
Sample of the information in ‘document format’ (http://iconsent.pilotvalidation.eu/wp-content/uploads/2020/04/Ingle%CC%81s-Adolescentes.pdf)

The sketches made by the children on the design of the website were taken into account for the visual and navigational design of the website. The website (Figure 3), offered the possibility of obtaining the information in the website, narrated video and/or written text (document in pdf format) with icons and images (Figure 4). At the bottom of the website, at the end of the information, the comprehension evaluation questionnaire was placed.

The final version of the *C‐CAsIn* for vaccine studies was designed in collaboration with the children in several sections:

(1) Introduction: explanation of the study, objective, procedure, duration of participation, right to withdraw, voluntariness, decision making
(2) Part A—Objective (Table 4): 14 items written in plain language, with two response possibilities symbolized by facial expressions and colours, green for agreement and red for disagreement. The questions tested comprehension of all sections of the information provided.
(3) Part B—Subjective (Table 5): 10 items whose wording starts with ‘I understood…’. The response possibilities are wider, with 5 possibilities between ‘I didn't understand anything’ and ‘I understood everything’. Also symbolized by a colour code and a visual facial code.
(4) The last section of the C‐CAsIn includes a short questionnaire with 8 items on sociodemographic data (age, sex and country of residence), previous experience of participation in a CT, the difficulty of the information received and preferred format and overall satisfaction with the information received.

The final digital assent form was created on a web page with a narrated video. All documents underwent several rounds of text adaptation, review of assent content requirements, review of the comprehension assessment tool, translation from Spanish into English and Romanian and linguistic adaptation for end users by native translators.

Finally, potential participants also tested the information prototypes, providing their final improvements which were taken into account before the information was uploaded to the target website and before validation in the target population of 620 children aged 12 and 13 in Spain, England and Romania.

Before final publication, it was checked that the recommendations for the preparation of the study information in the i‐CONSENT guidelines had been followed (Table 1).

It should be noted that the sessions did not seek consensus, but took into account all ideas and positions expressed in the design of the materials. Priority was given to suggestions that were common to the majority of participants.

The final materials are available on the following websites:

(1) Spanish version: http://iconsent.pilotvalidation.eu/estudio-adolescentes/;
(2) English version: http://iconsent.pilotvalidation.eu/en/teenagers-study/;
(3) Romanian version: http://iconsent.pilotvalidation.eu/ro/studiu-pentru-adolescenti/.

## 4 DISCUSSION

The process of designing the information materials for an ICP is perhaps the central part of any research study since it determines the potential participants' understanding of the information and, therefore, their autonomy in making free and informed decisions. This is also important to make the study population feel that they are at the centre of the research and that they participate and collaborate consciously and voluntarily.

There are various factors that influence the understanding and interpretation of the information a person receives, but it is the task of sponsors, industry and researchers to ensure that each and every participant understands it. The amount of information received by children before participating in a CT is overwhelming, as was seen in the two design thinking sessions conducted in this study. But, according to Regulation (EU)536/2014,^17^ it should include the nature, objectives, benefits, implications, risks and inconvenience of the CT, rights and guarantees of their protection, right to withdraw at any time without any problem and without justification, the conditions of the study, including the duration of participation and treatment alternatives. Faced with this large amount of information, the proposal developed in this study is to use a layered approach to present it. The first layer would contain brief information on the aspects covered by the legislation, and the second and successive layers would allow for further information. In this way, the child who wishes to know more about a specific aspect can expand on this information.

All this information should be clear, concise and adapted to the child's capacity to understand, but little account is taken of the information that children really want and need to know, as Roth‐Cline and Nelson^26^ pointed out. The systematic review carried out by Fons‐Martínez et al.^27^ shows that information needs are not the same for legislators, children, their parents and members of the research team. Focusing attention on the needs of children, it is observed that their interest is especially directed towards procedures, confidentiality and benefits^28^; knowing why they have been chosen to participate and if other children like them have already participated to ask them about their experience.^29^ In the study conducted by Tait et al.,^28^ slight differences were found with respect to gender at ages 13–17, with girls showing more interest in obtaining more detailed information about the procedure, objective, benefits, voluntariness and right to withdraw, and boys more interest in the alternatives.

But the amount of information is one thing; the difficulty of reading and understanding it is another. The urgent need to improve the readability of the information a minor receives before giving consent was already highlighted by Grootens‐Wiegers et al.,^30^ following a systematic review where the gap between the readability of the information and the reading level of minors was observed. Documents are often long, their readability low^31^ and the language complex, negatively impacting the ICP.^32^ What may seem simple to read and understand for trial sponsors and researchers can be complex for participants. In the present study, the readability of the initial information was improved by constructing shorter sentences with simpler terms, fewer syllables and more direct grammatical structures.^33^, ^34^ In this process, the contributions made by the children were of great help, as they participated in the drafting of the aspects that were more difficult for them to understand after being explained by the researchers.

To facilitate reading, the text was accompanied by simple pictures which, although not proven to significantly improve comprehension of the information, do improve satisfaction and the child's subjective belief that their understanding is improved.^35^

Attempts to improve the formats of information materials presented to children participating in research have been numerous in recent years, but none of them conclusive. Although the improved readability of written text and the comic format were shown to improve the comprehension of some aspects of the information presented to children compared to a traditional text format,^36^, ^37^ children participating in our design thinking sessions preferred other more interactive formats. The video format and the combination with multimedia tools^18^ have also shown improvements in understanding and satisfaction with the information received by children in numerous previous studies,^38^, ^39^, ^40^, ^41^ as preferred by the children who participated in the co‐creation process of the present study.

It is possible that all of these novel proposals in previous studies would have shown a greater positive impact on children's understanding and acceptance if they had also been involved in the design process.^13^ In this way, the information and format would have been better adapted to their needs and preferences. It is not about offering a wide variety, but about offering what each age group prefers. Even making information more readable and attractive to children does not ensure that they will understand it.

One of the fundamental problems is the lack of validated tools to assess the comprehension of information in minors participating in an assent process. Although it is best to assess the level of comprehension of information through a natural conversation between the potential participant and the researcher,^42^ these tools make it possible to homogenize the process of verifying comprehension, provide an objective record of comprehension during the assent process and serve as a support for those researchers who are less skilled in carrying out this assessment through a natural conversation. Several studies have developed and validated tools, such as the MacArthur competence assessment tool for clinical research (MacCAT‐CR)^43^ to assess the competence of minors, and the QuIC,^20^ which measures comprehension objectively and subjectively, in cancer patients involved in CTs. Other studies such as Chaisson et al.'s,^44^ Lee et al.'s^45^ and Blake et al.'s^46^ have developed ad‐hoc questionnaires with true/false items, to measure comprehension improvement after an intervention; none of these tools have been validated.

Based on the QuIC, as it is the most widely used questionnaire in different studies to measure comprehension, we adapted and created a new version for children, with the children's participation. Their participation at this point was crucial, as all their contributions to the items and the presentation format resulted in a new questionnaire (C‐CAsIn) that was shorter, more comprehensible and simpler in its response format.

Co‐creating by involving children increases the complexity of the process of designing information materials, but the benefit for them is direct, as it is adapted to their needs, increases their understanding and autonomy and therefore improves the decision‐making process.

The limitations found in the present study were related to the fact that the children were not real participants in the CT for which the materials were being developed, which could generate a bias in their response. Working with a sample of children living in the Valencian Region may affect the transferability of the results.

## 5 CONCLUSION

This article describes the methodology for the design and elaboration of IC materials for CTs with children (assent) and defines the specific tools to be used.

To ensure that the informational materials are tailored to the child's maturity, preferences and needs, it is recommended that a representative group of the target population be included in the design of the materials.

The design thinking methodology has proven to be an easy and useful tool to involve children in the design of information adapted to their needs and preferences.

It is recommended to conduct two working sessions focusing on three main topics:

1. what information is relevant to them;
2. which concepts are difficult for them to understand and
3. in what format they prefer to receive this information.

This will improve their understanding and promote their autonomy.

In addition, as part of the assent process in a CT, it is necessary to confirm that the information provided to the child has been understood. The C‐CAsIn survey has been designed, together with the children, to test understanding of information in the assent process of vaccine CTs, however, it should always be checked for its suitability to the particular study design.

## CONFLICT OF INTEREST

The authors declare no conflict of interest.

## Data Availability

The data that support the findings of this study are available on request from the corresponding author. The data are not publicly available due to privacy or ethical restrictions.
